# Training in the speciality of General and Family Medicine in Angola: A cross-sectional study

**DOI:** 10.4102/phcfm.v17i1.4812

**Published:** 2025-06-20

**Authors:** Clara T.L. da Silva Fernandes, Isabel N. da Mata Ferreira, Manuel F.D. dos Santos, Paulo Santos

**Affiliations:** 1 Department of Community Medicine, Information and Health Decision Sciences (MEDCIDS), Faculty of Medicine, University of Porto, Porto, Portugal; 2 Department of Surgery, Faculty of Medicine, Agostinho Neto University, Luanda, Angola; 3 Center for Health Technology and Services Research (CINTESIS@RISE), Faculty of Medicine, University of Porto, Porto, Portugal

**Keywords:** quality of training, SWOT analysis, primary health care, family practice, education, medical, graduate, physicians, primary care, health workforce, Angola

## Abstract

**Background:**

Primary healthcare plays a crucial role in health system, acting as the first line of assistance in preventing, treating and caring for diseases. In Angola, primary healthcare is a recent and developing reality.

**Aim:**

To evaluate the strengths and weaknesses of the Angolan General and Family Medicine speciality training programme, identifying areas for improvement and promotion of medical education quality.

**Setting:**

Primary healthcare doctors in Angola, including General and Family Medicine specialists and residents in training.

**Methods:**

A cross-sectional study was conducted from April 2024 to June 2024 using an online structured questionnaire. The survey was distributed via email and messaging platforms to all primary care doctors practising in Angola. Participants were asked about their opinions regarding the education process and training conditions. Two open-ended questions complemented the data collection.

**Results:**

A total of 584 doctors responded (61.1% females), with a mean age of 40.6 years. The most positively evaluated dimensions were faculty and mentoring, supervision, resident guidance, and programme evaluation. Conversely, the quality of infrastructure and access to educational resources were identified as major weaknesses in the training process.

**Conclusion:**

Despite limitations in teaching materials and infrastructure, the overall perception of General and Family Medicine training in Angola is positive. There is a recognised opportunity to expand and strengthen the programme nationally.

**Contribution:**

These findings reflect the perspectives of primary care doctors in Angola and provide valuable insights for policymakers and medical institutions to reinforce a speciality essential to national health system development and population health outcomes.

## Introduction

Primary healthcare (PHC) plays a crucial role in the health system, acting as the first line of defence in preventing, promoting and treating diseases. In Angola, a developing country with an evolving health system, PHCs are especially important in meeting the population’s health needs. The lack of resources and adequate infrastructure significantly challenges them.

Alma-Ata conference was established in 1978, and the importance of primary healthcare in achieving health for all is understood as complete physical, mental and social well-being and not just the absence of disease or infirmity.^1^ Since then, most countries have established this organisation in each health system.^2,3^

The 2024 World Health Statistics report of the World Health Organization (WHO) shows a significant increase in the Universal Health Coverage Score from 45 to 68 between 2000 and 2021 globally, although some stagnation during the coronavirus disease 2019 (COVID-19) pandemic, proving these efforts. Unfortunately, this is less visible in African countries, which have a considerable margin of progression in this goal.^4^

One of the problems is the need for more human resources. The number of primary care physicians and specialists in General and Family Medicine is associated with higher coverage, better health outcomes, lower overall costs and decreased preventable mortality.^5^ There is a relative shortage of doctors in rural areas of Africa because of limitations in the availability of educational resources despite the call for attention at every Regional Meeting of Africa of the World Organization of Family Physicians (WONCA). The proposed creation of a Faculty of Family Medicine in East, Central and Southern Africa may help by providing differentiated professionals adjusted to the specific regional needs, educating a new generation of doctors and providing a new way of practising in primary healthcare reality.^6^ The recent creation of the East Central and Southern Africa College of Family Medicine within the East, Central and Southern Africa Health Community will help provide differentiated support.

### The Angolan case

Angola Constitution affirms health as a fundamental human right. The current process of political and administrative decentralisation and the commitment of the government to accelerate the improvement of the health status of populations coincide with the definition of the municipality as a base unit for planning, organising and implementing primary health care services, including their articulation with the secondary (provincial) and tertiary (national) reference and counter-reference levels.^7^

Primary health care has been included as part of the National Health Service (NHS) Model since 1992 by Law 21-B/92, which designs the basis of the National Health System. It manages the most extensive public network of health services, dependent on the Health Ministry of Angola. The operationalisation of this decision occurred only in 2003 by the Presidential Decree 54/03, of 05 August 2003, which regulates the NHS Sanitary Units. Now, the public NHS ensures medical assistance through universal accessibility, equity, solidarity, social justice, quality, effectiveness and efficiency.

Primary Health Care is the essential care of the entire network of the National Health Service, acting in the coordination and integration of care to individuals and communities. They work cooperatively with the municipalities through the community and health development agents (ADECOS: *Agentes de Desenvolvimento Comunitário e Sanitário*), developing the Social Consultation Councils of the Municipality and the Municipal Health Management Teams, which guarantee the institutional participation in decision-making about the people’s health conditions.^8,9^

### Organisation of the National Health Service in Angola

The NHS is part of the National Health System. It covers all official institutions and services providing health care dependent on the Health Ministry, constituting the most extensive health network in the country. The NHS is administered at the provincial level by the Provincial Governments and Municipal Administrations.^9^

The National Health Policy of Angola establishes three hierarchical levels of service provision. Primary health care is at the ground level, located near the population, and is directed to people’s needs. It aligns with the PHC principles of equity, guaranteeing universal coverage and adequate access to health services, comprehensiveness, providing integral, integrated and appropriate care throughout the individual’s life, proactivity, with the emphasis on health promotion and disease prevention, and empathy, ensuring the first contact of the individuals, their families and community with the system and the right pathways to achieve the desired outcomes.

The Municipal Health System represents Angola’s NHS locally, serving as the first level of integrated individual and collective care.^10^ It involves several actors in promotion, prevention, treatment, rehabilitation and surveillance, acting to coordinate health promotion and addressing social determinants like sanitation, food safety, clean water, energy, education and leisure.

The Primary Healthcare-based Municipal Health System covers a defined population within a specific area, allocating resources for public and private healthcare, referral hospitals and support services like diagnostics and logistics. It ensures patient-centred care, accessibility and continuity to the community.

### History of General and Family Medicine in Angola and the importance of training

Following the consensus statement of the 2nd African Regional WONCA Conference,^11^ the speciality of General and Family Medicine in Angola was implemented on 13 May 2013, answering directly to the constitutional compromise of providing universal and free primary care to all. The Health Ministry recognised the formative suitability of the Multiprofile Clinic for training the first residents, which began in November of the same year. Its college was then constituted on 01 November 2019, by these first General and Family Medicine specialists from Luanda, Angola.

In 2021, in collaboration with the Medical Council of Angola, the Angolan Ministry of Health launched the National Family Medicine Expansion Program as a long-term strategy for providing, securing and training family doctors in community health centres. Three main aspects make this National Program unique in the Angolan context^1^: the joint effort and engagement of the Ministry of Health with the Medical Council of Angola and local health authorities in the design and implementation of this programme^2^; decentralisation of training sites, with residents in all 18 provinces, including rural areas and^3^ use of community health centres as the principal place of practice and training.

Primary Health Care plays a crucial role, with family doctors as the system’s cornerstone, ensuring accessibility, continuity and patient-centred care. Despite limited resources, they help reduce disparities and manage conditions through screening, diagnosis, treatment and follow-up.^12^

In Angola, General and Family Medicine training began only 11 years ago, and there are still struggles for recognition within the health system, among specialists, and the public. High-quality education and training are essential to expanding the workforce, ensuring national coverage and maintaining care standards. Strengthening this field is key to a more effective and sustainable healthcare system.

The College of Family Physicians of the Medical Council of Angola developed the training programme. The curriculum at the Multiprofile Clinic served as a pedagogical basis for the National Program. Adaptations have been made for less favoured provinces with the mandatory rotations and minimum required curriculum that a resident must experience throughout the course. Finally, the Ministry of Health of Angola decided to implement the programme in all municipalities with the primary objective of providing medical coverage in all municipalities of the country.

Cuban doctors from the Cuban cooperation with Angola were allocated to each province to supervise three to six residents in their daily clinical activities. Like in Cube and many other countries, general and family physicians are expected to be the first contact clinicians at the community health centre level, making this contribution particularly relevant for the training of our new specialists. Over the 3 years of training, residents completed rotations lasting 4 to 6 weeks in various areas. Most of your training occurs at the community health centre.^13^

Currently, Angola has 352 specialists in General and Family Medicine (151 Angolans, 170 Cubans and others) and 600 residents distributed for several years of education and training.

Primary Health Care and family doctors are intrinsically associated because they are the first-line doctors. The quality of training in this speciality will impact the quality of the PHC of a country by better managing the risk factors and diseases and reducing the consumption of hospital and tertiary-level care. The training of family doctors is then an investment in PHC and people’s health.

We aim to analyse the conditions of education and training of General and Family Medicine residencies in Angola, prospecting for their strengths and weaknesses, allowing us to characterise the threats and opportunities to improve the quality of training and, thus, the quality of Primary Healthcare in our country. Understanding the current situation would allow the implementation of quality improvements in public policies, local processes and individual efforts to improve health services in Angola.

## Methods

### Type of study

We performed a cross-sectional study based on a survey that included doctors working in primary health care in Angola.

### Population and setting

All 852 doctors, including specialists in General and Family Medicine, teachers, monitors and residents working in Primary Health Care in Angola, were invited to participate, including all 18 provinces: Benguela, Bié, Bengo, Cabinda, Cunene, Cuando-Cubango, Cuanza-Norte, Cuanza-Sul, Huíla, Huambo, Luanda (the capital), Lunda-Norte, Lunda-Sul, Malange, Moxico, Namibe, Uíge and Zaire. Invitations were distributed by e-mail and personal messages and repeated several times between April 2024 and June 2024.

### Sources of information

A structured electronic questionnaire was built for this survey using Google^®^ forms. Six dimensions were included for analysis: quality of infrastructure and resources, faculty and mentoring, curricular structure and pedagogical approach, supervision and guidance of students, practical exhibition and learning experiences and feedback and evaluation of the programme.

We used a Likert scale from 1 (very insufficient) to 7 (excellent) points for each question. Each Likert scale was categorised as insufficient (1–4) and good (5–7) quality appreciation for analysis. The questionnaire also included a vision of the current speciality situation, specific training and geodemographic characterisation.

Two final open-ended questions asked for three aspects of the training programme considered relevant and valuable for the training programme (positive aspects) and another three aspects that need improvement (negative elements). Answers were analysed after categorisation in the main topics and organised by the order of relevance for each participant. The questionnaire was previously tested on a small number of respondents not included in the sample to verify the questions’ comprehensibility and face validity.

The total satisfaction score consisted of the sum of the structured questions, divided by the median, representing the most satisfied and those least satisfied.

### Statistical analysis

The Excel^®^ database was translated for the SPSS® version 28 programme for analysis. We used descriptive analyses (proportions or mean, according to the variables) to describe the categorical variables and mean and standard deviation for continuous variables. Confidence intervals were calculated using the Wald test. Logistic regression allowed us to calculate the odds ratio (OR). The significance level adopted was 5% (*p* < 0.05).

### Ethical considerations

The Director of the Institute of Health Expertise in Angola approved the project. All procedures followed strictly ethical principles, according to the Helsinki Declaration. The participants included in the study consented to participate in the research by accepting the Digital Informed Consent Form.

## Results

The total number of participants was 584 (68.5% of total physicians invited), with a mean age of 40.6 years (61.1% females). The majority were Angolans (79.8%) and worked in the public system (89.0%). Specialists represented 31.1% of the total participants. The main regions were Luanda (22.3%), Huambo (11.5%) and Uíge (9.6%), as expected by the national distribution of physicians (Table 1). Participants had an upbeat assessment of the speciality of General and Family Medicine in the country (84.1%), considering that it presents good quality (72.9%) and good education and training (78.8%).

**TABLE 1:** Characterisation of the participants.

Variables	Frequency		%
	*n*	*N*	
**Sex**			
Male	227	584	38.9
Female	357	584	61.1
**Age†**			
Less than 40 years	335	579	57.4
Greater than 40 years	244	579	41.8
**Country of origin**			
Angola	466	583	79.9
Cube	116	583	19.9
Mozambique	1	583	0.2
**Province of training**			
Luanda	130	582	22.3
Huambo	67	582	11.5
Uíge	56	582	9.6
Cabinda	51	582	8.7
Huíla	51	582	8.7
Malange	44	582	7.5
Benguela	20	582	3.4
Cuanza sul	18	582	3.1
Lunda-Sul	18	582	3.1
Bié	16	582	2.7
Cuando-Cubango	14	582	2.4
Moxico	13	582	2.2
Cuanza-norte	12	582	2.1
Lunda-norte	10	582	1.7
Zaire	10	582	1.7
Bengo	5	582	0.9
Cunene	4	582	0.7
Other	23	582	3.9
**Institution funding**			
Public	520	584	89.0
Private	27	584	4.6
Mixed	37	584	6.4
**Status**			
Specialist (GFM)	183	584	31.3
Non-specialist	401	584	68.7
**Quality of the current state of GFM speciality in Angola**			
Good quality	426	584	72.9
**Quality of the current state of GFM training in Angola**			
Good quality	460	584	78.8
**Quality of the general assessment of the GFM speciality in Angola**			
Good quality	491	584	84.1

GFM, general and family medicine.

† , Mean (±DP) 40.7 ± 9.2.

The dimensions of the training programmes that were best evaluated were the faculty, mentoring, supervision and residents’ guidance, with mean proportions of good evaluation above 80%, followed by feedback and evaluation of the programme. The main constraints were identified in the quality of infrastructure and resources (Table 2).

**TABLE 2:** Proportion of participants who positively evaluate the different aspects of medical training in General and Family Medicine in Angola.

Aspects of medical training	*n*	%	95%CI
**Quality of infrastructure and resources**			
Quality of the physical facilities used in the training programme	358	61.3	57.4–65.3
Access to libraries	150	25.7	22.0–29.3
Access to laboratory	159	27.2	23.6–30.9
Access to printed learning material	218	37.3	33.5–41.4
Access to information technology	275	47.8	43.1–51.3
Access to databases and online resources	279	47.8	43.7–51.8
**Faculty and mentoring**			
Quality of teachers and mentors involved in the training programme	521	89.2	86.6–91.7
Commitment and motivation of teachers and monitors in teaching and guidance	515	88.2	85.5–90.7
Integration and individualised guidance with teachers and monitors	517	88.5	85.8–91.0
**Curricular structure and pedagogical approach**			
Organisation and structure of the training programme curriculum	488	83.6	80.8–86.8
Teaching methodology used to promote learning and development of practical skills	476	81.5	78.6–84.9
Curriculum structure to assess the health needs of the local population	363	62.2	58.4–66.3
**Supervision and guidance of students**			
Supervision received during your practical and clinical activities	478	81.8	78.8–85.1
Support to deal with challenges and doubts during the learning process	474	81.2	77.9–84.2
**Practical exhibition and learning experiences**			
Variety in learning	384	65.8	62.1–69.8
Quality of experiences in practice	414	70.9	67.1–74.5
Preparation to deal with real clinical cases	433	74.1	70.8–77.9
**Evaluation process**			
Feedback and evaluation of the programme	487	83.4	80.4–86.5
Transparency of the process	409	70	66.5–74.0
Relevance of the evaluation	419	71.7	68.1–75.4
Effectiveness for professional development	443	75.9	72.4–79.4

Age, time of medical exercise, working in a private institution and being connected to teaching were associated with a good evaluation of General and Family Medicine training, while Angolans presented lower satisfaction. A good appreciation of the training conditions was associated with a positive view of the speciality and its current state in exercise and training. In the multivariate analysis, only age is associated with satisfaction with training conditions (OR = 1.037; 95%CI: 1.014–1.059; *p* < 0.001), with younger doctors presenting greater dissatisfaction with training (Table 3).

**TABLE 3:** Impact of sociodemographic characteristics on satisfaction with General and Family Medicine training.

Sociodemographic characteristics	OR	95%CI	*p*-value*
Sex female (vs male)	1.25	0.89–1.74	0.196
Age	1.03	1.02–1.05	< 0.001
Angolan nationality	0.54	0.36–0.82	0.004
**Institution funding**			
Public	<ref>	-	-
Private	9.12	3.18–26.11	< 0.001
Public-private	2.89	0.75–11.12	0.123
Time of exercise as a doctor	1.04	1.02–1.06	< 0.001
Time of exercise in the speciality	1.01	0.99–1.03	0.271
Specialists (vs residents)	0.80	0.56–1.14	0.212
Teachers	1.97	1.06–3.65	0.032

GFM, general and family medicine; OR, odds ratio.

* , Logistic regression.

The two last questions were open ended. All answers were manually collected, analysed and categorised. The most highlighted topics were the clinical experience and practice, interaction with the community and professional development. The aspects pointed out as negative to improve were the focus on Primary Health Care and curriculum improvement, better working conditions and education, institutional support and appreciation of the speciality (Table 4).

**TABLE 4:** Proportion of participants who identified relevant and improvement aspects in the General and Family Medicine training programme.

Aspects of the General and Family Medicine training programme	Proportion (%)	95%CI
**The most relevant and valuable aspects of practical experience during the training programme**		
Experience and clinical practice	47.8	43.6–52.0
Interaction with the community	33.8	29.9–37.8
Professional development	30.2	26.4–34.1
Education and training	29.7	25.9–33.5
Reviews and improvements	12.3	9.6–15.1
Impact and motivation	12.3	9.6–15.1
Challenges and needs	10.9	3.3–13.5
Infrastructure and resources	8.2	5.9–10.5
Human and ethical aspects	6.8	4.7–08.9
**Aspects that need improvement in the training programme**		
Focus on Primary Health Care and curriculum improvement	62.9	58.9–66.9
Better working and teaching conditions	41.6	37.5–45.7
Institutional support and enhancement of the speciality	20.2	16.9–23.5
Increase the number of teachers and monitors	18.1	14.9–21.2
Monitoring and continuous follow-up	17.5	14.4–20.7
Support and encouragement to the interns	10.9	8.3–13.4
Integration and inter-institutional collaboration	5.8	3.9–7.7

## Discussion

Since the Alma Ata conference in 1978, the importance of Primary Health Care has been well established to provide medical care at different levels of intervention to the population, maximising the universal right to health from a perspective of equity and with sustainable costs.^1,14^

The training of doctors in General and Family Medicine is crucial to achieving this objective because of the possibility of disseminating them throughout the territory and providing local, comprehensive, continuous and coordinated care to the entire population.^15^

In Angola, this speciality is recent and appears under the recommendations of the 2nd African Regional WONCA Conference of 2009,^11^ adopting the most common nomenclature in Portuguese-speaking countries.

This study highlights the fundamental aspects of the education and training in General and Family Medicine in Angola. It is innovative in its approach and conclusions, allowing us to strengthen our country’s entire training and educational process. Better training in General and Family Medicine will improve medical practice and impact the health of all Angolans.

### Evaluation of the different aspects of medical training in General and Family Medicine in Angola

Globally, training in General and Family Medicine in Angola presents a good faculty that is highly committed and of good quality, offering continuous supervision during practical and clinical activities, adequate support in dealing with challenges and doubts and good feedback and evaluation. The curriculum structure and teaching methodology are also strong points. In recent years, international trainers with high qualifications have guaranteed postgraduate education and specialisation in Angola, mainly from Cuba.

With the expansion of the speciality at the national level, the Ministry of Health of Angola, in collaboration with the Medical Council, launched the National Program for the Expansion of General and Family Medicine. This programme aims to increase the number of family doctors in community health centres. It is supported by cooperation agreements between Angola and Cuba, which have a long history of collaboration, especially in health. Cuba, known for its robust Primary Healthcare System, offers experienced doctors to support the training and delivery of medical services in Angola. Despite the increasing number of Angolan trainees, they are still very few to ensure the necessary internal autonomy.

The limitations are identified mainly in the infrastructures with few or poorly equipped laboratories; scarcity of updated printed material; limitation of interinstitutional partnerships; poor quality Internet access and lack of adequate libraries, print or digital. This challenging context is reflected at the national level but is more acute in the provinces with fewer resources. These constraints are still sequels from the long period of civil war, with a deviation of funding from health to other priorities, which are now urgent to correct. In recent years, Angola has been increasing the number of health infrastructures to meet the number of inhabitants. Since 2009, the Integrated Plan for Intervention in Municipalities^9^ intends to increase the autonomy of the 164 municipalities, aiming to improve the quality of life throughout the national territory through policies of dissemination and decentralisation of administrative powers. Interconnecting with this problem, we also point out the lack of adequacy of the curriculum to the local needs of the population. Primary Health Care is a discipline of proximity. Social, geographical and community characteristics,^16^ such as rural areas without a sanitary infrastructure or potable water distribution, long distances to medical care, specific epidemiology of diseases or lack of adequate resources, both in ambulatory and in hospitals, are crucial for the provision of care and should be incorporated into the medical training in Primary Healthcare.^1,14^ The Angolan population, especially in rural and peripheral areas, faces significant challenges in accessing health services because of a lack of infrastructure, resources and qualified professionals. Angolan physicians must be prepared for the Angolan situation rather than import international models, which may be suitable for other countries but unsatisfactory for our population.

### Socio-demographic characteristics

The main predictors of higher satisfaction with General and Family Medicine training in Angola are age, with older slightly more satisfied than younger ones, experience as a doctor, working in a private setting, and teaching. On the other hand, younger and Angolan nationals are less likely to be satisfied. A positive perception of the General and Family Medicine speciality in Angola and the current state of the speciality and its training are strong predictors of higher satisfaction. These results are quite expected as residents are younger, have a higher workload and are often away from their homes. On the other hand, Angolans and Cuban specialists show higher satisfaction, which may be justified because having the title allows them to have higher salaries, recognition and career progression.

In general, as in society, the younger are for change and the oldest for maintaining status. Angola presents a great opportunity at this point. The mean age of Angolan specialists is relatively low, representing the new generation arriving in medicine. This may be a point of change towards quality and rigour framed by ethical and professional experience from those in charge.^17^

Luanda province has the highest concentration of family doctors, specialists and residents, which is expected, as it was the first province to implement the speciality in 2013 through a pilot project. On the other hand, Luanda is the capital of Angola, where there is better infrastructure and a more significant number of qualified teachers. Many doctors come to Luanda to do their training and do not return home after that. We included participants from the whole country to get their impressions and to frame the heterogeneity of working in different places. However, the total number does not permit a complete statistical evaluation.

The training of General and Family Medicine specialists is a national effort to strengthen the medical care of all Angolans. Despite the importance of the capital, the country needs to have good healthcare facilities, including human resources, in all provinces. As proximity is one of the main characteristics of Primary Health Care and a guarantor of equity, this topic becomes even more critical when we think about the curricular structure and the need to train residents for the different scenarios they will potentially face as specialists. Nevertheless, doctors cannot be required to work without decent working conditions to achieve effective health results.

### Overview of the General and Family Medicine speciality and training in Angola

The participants’ views on the speciality and training in General and Family Medicine were largely positive, which means high hope for the future. In Box 1, we propose a SWOT analysis based on our results as a reflection of the stabilisation of the strengths and the search for solutions to the weaknesses, considering the opportunities and the threats we found.

BOX 1 SWOT analysis.

**Strengths**

**1. Qualified and committed faculty:**

The General and Family Medicine training has highly qualified teachers, many of them of Cuban nationality, with experience and commitment to medical education.

**2. Structured programme and practical involvement:**

The curriculum is considered well structured, focusing on developing practical skills and clinical experience, providing a solid foundation for physicians in training.

**3. International collaboration (Angola-Cuba):**

Cooperation between Angola and Cuba has been critical in General and Family Medicine training, providing support through qualified teachers and experienced doctors.

**4. Positive assessment by participants:**

Despite the difficulties, the doctors in training and specialists have shown a positive evaluation of the speciality and teaching, evidencing a favourable climate for developing General and Family Medicine.

**Weaknesses**

**1. Insufficient infrastructure and resources:**

The need for libraries, laboratories and printed teaching materials was identified as a significant weakness, limiting students’ teaching quality and practical experience.

**2. Limited access to teaching materials and technology:**

Access to the Internet and scientific databases is scarce, especially in rural areas, which makes it difficult for doctors in training to research and update their knowledge.

**3. Curriculum adjusted to the needs of the population:**

The curriculum needs adjustments to reflect better the population’s specific needs, especially in primary healthcare, where the demand for general practitioners is higher.

**4. Insufficient valuation of the speciality:**

General and Family Medicine still faces challenges of recognition and appreciation by other medical specialities and the population, which can affect professionals’ motivation.

**Opportunities**

**1. Expansion of the speciality nationally:**

The National General and Family Medicine Expansion Program offers a unique opportunity to increase the number of family doctors and trainers in the country and to strengthen the speciality, especially in the most needed areas.

**2. Partnerships with international institutions:**

International partnerships can be expanded to strengthen training through academic exchanges, infrastructure development or access to international materials and databases.

**3. Use of distance learning technologies:**

The development and implementation of distance learning platforms can help overcome geographical and infrastructure limitations, allowing doctors in rural areas access to quality resources.

**4. Strengthening Primary Health Care:**

With the growing global emphasis on Primary Health Care, there is an opportunity to consolidate General and Family Medicine as a critical piece of the Angolan health system, training doctors to act on the front line of prevention and community care.

**Threats**

**1. Lack of financial and material resources:**

The lack of continuous investments in health and education can compromise the improvement of infrastructures and access to critical resources for medical training.

**2. Regional inequality of infrastructure:**

The disparity between infrastructure in urban and rural areas can result in unequal training, with doctors in more peripheral regions facing more significant difficulties in acquiring quality training.

**3. Lack of incentives for professionals:**

The lack of institutional and financial incentives may discourage doctors from remaining in the speciality, especially in rural areas where working conditions are more challenging.

**4. Dependence on foreign doctors:**

General and Family Medicine training still depends heavily on foreign doctors, which can become a threat in the long run if there is no effective plan to train and fix more national experts.

SWOT, strengths, weaknesses, opportunities, threats.

The aspects pointed out are fundamental in speciality training in Angola. They reflect the reality of medical practice in the country, where family doctors must be adaptable, versatile and highly qualified regarding technical skills and patient relationships. These factors are crucial to ensure that trained physicians are prepared to respond to the population’s needs, particularly in the most vulnerable and hard-to-reach regions.

Although General and Family Medicine have progressed considerably, they still face challenges related to recognition, both by other medical specialities and the population itself. Being a relatively new speciality, its full integration into the Angolan health system requires more time and effort to consolidate its importance in the country’s health landscape. A structured training programme is considered a substantial benefit for the training of these doctors. However, there is ample space for improvement as they focus on the need to give high importance to curricular development and its adaptation to the reality of the Angolan health system. What functions well in the capital, Luanda, does not necessarily fit other minor and more rural provinces, calling for a local adjustment according to the specific characteristics. Nevertheless, the time is now: there are conditions and will to advance, and the way will be constructed with each additional step we take.^13^

### Critical analysis

The elaboration of this study was challenging on several fronts. The slow authorisation for the study in Angola was an initial obstacle that delayed the progress of the research. In addition, professionals’ awareness of answering the questionnaires was another significant barrier, especially in rural regions where access to the Internet is minimal. These limitations, combined with the fact that General and Family Medicine is a new speciality in the country and with few reference articles for comparison, made the research process more arduous and more relevant in our reality.

Despite the difficulties, we received many responses from all over the country, and a relevant depth of information was collected. Our data provide a broad and representative view of General and Family Medicine training in Angola and a solid basis for future improvement initiatives.

### Future directions

Our results reflect the thinking of General and Family Medicine doctors in Angola. They guide responsible institutions from government and medicine to take action and improve education programmes and allocate resources for training to reach high-quality standards and excellence in practice for a better healthcare system in our country. In this study, we close some conclusions, and, at the same time, we open many more questions about the opportunities to improve the education and training of general and family physicians in Angola. Many questions need a specific approach by continuing this research to obtain more granular, qualitative responses to guide further educational improvements.

The goals are pretty simple: infrastructure improvement, access to teaching materials and resources, the adaptation of the curriculum to the population’s needs and the improvement of the appreciation and awareness of the speciality. Several strategies may help to achieve them: creating a national family doctor campaign to raise awareness among the population and health professionals about the crucial role of these providers; increasing the investment in medical education, namely in international partnerships to promote academic exchanges, teacher training and infrastructure development and expanding the programmes in rural areas, creating incentives for physicians in training to be allocated in rural areas and ensure they have access to resources for their continuing education.

## Conclusion

The study of General and Family Medicine training in Angola reveals a positive panorama in terms of the quality of the teaching staff, which is composed of highly qualified teachers committed to medical education. However, the research also highlights areas that need attention, such as improving teaching infrastructures, access to learning materials and resources and adapting the curriculum to the real needs of the Angolan population.

Training physicians to act on the front line of the health system, focusing on prevention and primary care, is fundamental. The dynamic nature of global health, marked by migratory movements, globalisation and the emergence of new diseases, makes it essential to prepare family doctors to face complex challenges and provide care that reflects the specific needs of the communities they serve. The strengthening of the curriculum, which is focused on primary health care and adjusted to the socioeconomic and geographical context of Angola, is one of the main priorities for the future of medical training in the country.
